# Future stroke risk in the chronic phase of post-percutaneous coronary intervention

**DOI:** 10.1371/journal.pone.0251253

**Published:** 2021-05-06

**Authors:** Shinsuke Muraoka, Daiki Somiya, Aoi Ebata, Yuki Kumagai, Naoki Koketsu

**Affiliations:** Department of Neurosurgery, Tosei General Hospital, Aichi, Japan; University of Tampere, FINLAND

## Abstract

A percutaneous coronary intervention (PCI) is widely performed for acute coronary syndromes or chronic coronary syndromes. Periprocedural stroke is a clinically significant complication during PCI. The incidence of cerebrovascular events (CVEs) after PCI in the chronic phase is obscure. This study aimed to investigate the prevalence of CVEs after PCI in the chronic phase and evaluate the usefulness of a simple coronary artery calcification (CAC) evaluation method. This prospective observational study included 179 patients who underwent PCI between January 2016 and December 2018. The incidence of cerebral infarction was examined from one month after PCI to December 2019. In total, 171 individuals (134 men; mean age, 69.8 ± 9.8 years) were recruited. During a median follow-up period of 33 months, the onset of cerebral infarction was observed in 20 individuals (11.7%). More CAC sites (p = 0.009) and post-PCI for the chronic coronary syndrome (p = 0.049) showed a significant association with future CVEs. There was no significant cervical internal carotid artery stenosis for patients who occurred CVEs. The cutoff value for the number of CAC sites for predicting future CVEs was 4.5. The new and easy method accurately reflected future CVEs risk and may be clinically applicable.

## Introduction

Millions of patients are treated with a percutaneous coronary intervention *(PCI)* each year for acute coronary syndromes or chronic coronary syndromes. Perioperative stroke during PCI is a clinically significant complication associated with high mortality and morbidity. The incidence of post-PCI cerebrovascular events (CVEs) in the acute phase is low. However, it has increased significantly over the last decade due to the increasing complexity of the patients being treated and PCI technology’s complexity. Notably, the incidence of CVEs after PCI in the chronic phase is about 1–11% [1–4], though the risk factors of post-PCI CVEs were not currently known well.

Excessive coronary artery calcification (CAC) can lead to adverse outcomes after PCI. CAC is also associated with the prevalence of clinically significant coronary atherosclerosis. Coronary computed tomography angiography (CCTA) has been used to assess coronary atherosclerosis using non-invasive measurements [5]. Agatston’s score is the most widely used method to assess CAC quantitatively [6,7]. Agatston’s CAC scores were calculated based on the number, areas, and peak Hounsfield computed tomographic numbers of the calcific lesions detected. This method was only used with specific application software.

The purpose of this study was to investigate the incidence of CVEs after PCI in the chronic phase and evaluate the usefulness of a more direct evaluation method for CAC compared to previously used Agatston’s methods.

## Materials and methods

This study was approved by Tosei General Hospital ethics committee on March 25th, 2020 (approval number: 863). The written informed consent was obtained from all participants.

### Study design and population

We prospectively included patients who underwent PCI at Tosei General Hospital between January 2016 and December 2018. The incidence of cerebral infarction was determined from one month after the date of PCI until December 2019. After PCI, all patients underwent CCTA examination. All patients underwent close follow-up at the outpatient clinics every 3–6 months, and the occurrence of any CVEs was recorded prospectively. In case of the occurrence of new neurological symptoms, patients received brain magnetic resonance imaging (MRI). When brain MRI revealed a new ischemic lesion, this patient was recorded as CVE positive, and cervical carotid artery sonography was performed. The exclusion criteria were as follows: acute or subacute CVEs (i.e., within 30 days of the PCI) and lack of follow-up data.

This study was approved by the institutional review board (the approval number: 863), and written informed consent was obtained from all participants.

### Clinical data collection

Demographic, medical history, and laboratory parameter data were obtained from medical records. Hypertension was defined as systolic blood pressure ≥140 mmHg or diastolic blood pressure ≥90 mmHg. Any of the following blood test results led to a diagnosis of diabetes mellitus: fasting blood sugar ≥126 mg/dL, or hemoglobin A1c ≥6.5%. Any of the following blood test results led to a diagnosis of dyslipidemia: low-density lipoprotein of >140 mg/dL, total cholesterol of >200 mg/dL, or triglycerides of >150 mg/dL. Current smoking was defined as smoking in the past 30 days. Body mass index was calculated as weight (kg)/height^2^ (m^2^).

### Imaging data collection

#### Computed tomography angiography

All patients underwent CCTA using a 320-row computed tomography scanner (Aquilion ONE Nature edition; Canon, Tochigi, Japan). A prospective electrocardiogram trigger axis scan was performed in all patients. The computed tomography data collection parameters were as follows: detector configuration, 320 × 0.5 mm; tube potential, 120 kVp. The automatic exposure control system for image noise automatically determines the optimum tube current that matches the target standard deviation value of 15.

Patients received 24.5 mg/kg/sec of iopamidol (Iopamiron 370, 370 mg/mL; Bayer, Osaka, Japan) to patients. A 20 mL saline flush was delivered at the same injection rate used for the contrast, followed by 11-second contrast injection. The bolus-tracking method was used in the following protocol: when the ascending aorta reached 170 Hounsfield units, patients were instructed to inhale and hold, at which point the scan was initiated. The forward-projected model-based iterative reconstruction solution cardiac sharp and strong algorithm was used for reconstruction. The slice thickness and increments used were 0.5 mm and 0.25 mm, respectively.

#### Sonography

Patients received cervical carotid artery sonography who had a new ischemic lesion in brain magnetic resonance imaging. A 5-MHz linear-array transducer was used as dictated by the patient body habitus. Patients underwent color and spectral Doppler imaging as well as grayscale. The angle adjustment was based on the flow direction indicated by the color Doppler. Angle-adjusted spectral Doppler samples were taken from a given site within each common carotid artery (CCA) and the ipsilateral internal carotid artery (ICA), containing the proximal, middle, and distal components of each vessel. The ICA’s maximum velocities observed within each proximal, middle, and distal segment was recorded by technologists. The highest values of these recorded velocities were routinely reported by interpreting radiologists as peak systolic velocities. Doppler parameters regularly evaluated and reported for each carotid bifurcation included peak systolic velocity, end-diastolic velocity, and the ratio of ICA’s peak systolic velocity to that of the ipsilateral distal CCA.

In patients with asymptomatic carotid artery stenosis of 50% or more, the incidence of ipsilateral stroke is 1–3% per year, and the incidence of immediate stroke or transit ischemic attack was 3–5% per year [8]. Observational studies of patients with asymptomatic carotid artery stenosis of 50% or more showed that antiplatelet drugs could reduce ischemic cerebrovascular events and cardiovascular disease [9,10]. In 1991, the North American Symptomatic Carotid Endarterectomy Trial demonstrated carotid endarterectomy’s effectiveness in patients with more than 50% symptomatic internal carotid artery (ICA) stenosis [11]. Peak systolic velocity >150 cm/s is one of the most reliable markers of more than 50% ICA stenosis [12,13]. In this study, significant ICA stenosis was defined as the degree of stenosis greater than 50% and was estimated from ultrasonographic data.

### Evaluation of coronary artery calcification

According to Agatston’s method, 20 contiguous 3 mm slices were obtained of the proximal coronary arteries using plain computed tomography. Agatston’s CAC scores were calculated based on the number, areas, and peak Hounsfield computed tomographic numbers of the calcific lesions detected [6].

In this study, CCTA images were evaluated to assess for CAC. The threshold for the lesion to be classified as calcified. It was set to a computed tomography density of 130 Hounsfield units. CAC positive was determined as the existence of the lesion over 130 Hounsfield units. CAC was assessed as calcification on the right coronary artery #1, 2, left main trunk artery #5, left anterior descending coronary artery #6, 7, or left coronary artery circumflex branch #11. The number of CAC sites (minimum 0–maximum 6) was recorded.

### Statistical analysis

We used SPSS for Windows version 20.0 (IBM Corp., Chicago, IL, USA) for all statistical analyzes. Data are expressed as percentages or means ± standard deviation (SD). Fisher’s exact test, χ^2^ test, and Mann-Whitney U test were used to evaluate risk factors for future CVEs. A forward stepwise logistic regression was used in model building with the interrogated parameters. In the final multivariate analysis, the statistical significance level was set at *P* < 0.05. We used receiver operating characteristic curve analysis to analyze age validity and the number of CAC sites. We used the Youden index to determine the cutoff values of age and the number of CAC sites that could predict the future occurrence of CVEs.

## Results

### Patient characteristics

A total of 171 cases (134 men; mean age, 69.8 ± 9.8 years; mean body mass index, 23.9 kg/m^2^) were recruited (Table 1). Atrial fibrillation was observed in 8 cases (4.7%), hypertension in 101 cases (59.1%), diabetes mellitus in 68 cases (39.8%), dyslipidemia in 157 cases (91.8%), and positive smoking history in 99 cases (57.9%). Blood test findings were as follows: TG, 144 [130–159] mg/dL; HDL-C, 51 [49–53] mg/dL; LDL-C, 100 [94–106] mg/dL; and HbA1c, 7.0 [6.0–7.9] %. Fifty-six patients received PCI for acute coronary syndrome (ACS), and 115 patients received PCI for chronic coronary syndrome (CCS). Antiplatelet therapy was performed for 113 patients before PCI and 169 patients after PCI, respectively.

**Table 1:** Baseline characteristics of the study population.

**Age, y, mean (SD)**	69.8 (9.8)
**Male (%)**	134 (78.4)
**BMI (95% CI)**	23.9 (22.3–25.5)
**Prior comorbidities**	
**Atrial fibrillation (%)**	8 (4.7)
**Hypertension (%)**	101 (59.1)
**Diabetes mellitus (%)**	68 (39.8)
** HbA1c, %**	7.0 (6.0–7.9)
**Dyslipidemia (%)**	157 (91.8)
** TG, mg/dL (95% CI)**	144 (130–159)
** LDL-C, mg/dL (95% CI)**	100 (94–106)
** HDL-C, mg/dL (95% CI)**	51 (49–53)
**Smoking history (%)**	99 (57.9)
**The reason for PCI**	
** ACS (%)**	56 (32.7)
** CCS (%)**	115 (67.3)
**Medications**	
**Antiplatelet drugs before PCI (%)**	113 (66.1)
**Antiplatelet drugs after PCI (%)**	169 (98.8)
**Anticoagulant drugs before PCI (%)**	8 (4.7)
**Anticoagulant drugs after PCI (%)**	18 (10.5)

BMI: Body mass index; TG: Triglyceride; LDL-C: Low-density lipoprotein cholesterol; PCI: Percutaneous coronary intervention; CI: Confidence interval; HDL-C: High-density lipoprotein cholesterol; SD: Standard deviation; ACS: Acute coronary syndrome; CCS: Chronic coronary syndrome.

### Coronary artery calcification

**Fig 1:**
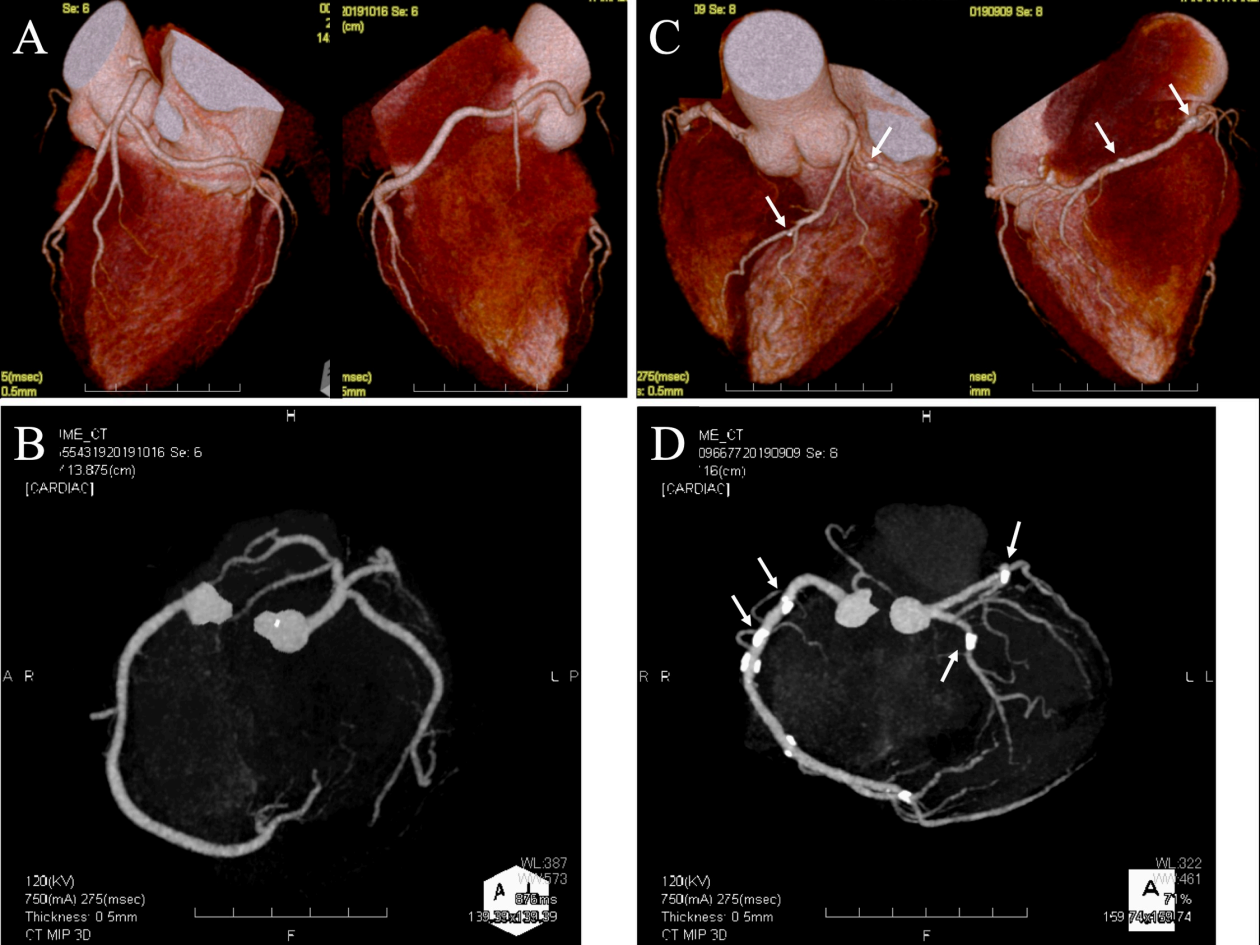
**Representative case.** (A) Three-dimensional (3D) reconstruction and (B) axial computed tomography image showing no coronary artery calcification (CAC). (C) 3D reconstruction and (D) axial image showing CAC; #1, #2, #6, and #11 are highly calcified. The number of CAC sites was 4 (arrow).

CCTA images were evaluated to assess coronary artery calcification. The number of CACs was counted (Fig 1). A list of coronary artery calcifications for each patient based on the American Heart Association classification was provided in the supplementary material. Coronary artery calcification of left main trunk (LMT) was observed 55 cases (32.2%).

During a median follow-up period of 33 months, the onset of cerebral infarction was observed in 20 cases (11.7%). The risk factors for future CVEs are shown in Table 2. Univariate analysis showed that CAC was significantly associated with future cerebral infarction (p = 0.015) and LMT calcification (p = 0.038). Higher age, male sex, hypertension, diabetes mellitus, smoking history, and atrial fibrillation were not related to the risk of future CVEs. According to multivariate analysis, more CAC sites (p = 0.009) and post-PCI for CCS (p = 0.049) were significantly related to the risk of future CVEs (Table 3).

**Table 2:** Risk factors of future cerebrovascular events on univariate analysis.

	Cerebrovascular events		*P* value
	Yes (n = 20)	No (n = 151)	
**Mean (SD) age, y**	73.0 ± 8.1	69.5 ± 9.6	0.129
**Male (%)**	16 (80.0)	118 (78.1)	0.850
**BMI (95% CI)**	22.8 (22.1–23.5)	24.1 (22.3–25.9)	0.609
**Hypertension (%)**	14 (70.0)	86 (57.0)	0.294
**Laboratory data**			
** HbA1c, %**	6.4 (6.2–6.6)	7.1 (5.9–8.2)	0.765
** TG, mg/dL (95% CI)**	130 (115–144)	146 (130–162)	0.392
** LDL-C, mg/dL (95% CI)**	99 (89–109)	99 (94–105)	0.918
** HDL-C, mg/dL (95% CI)**	49 (47–52)	53 (50–55)	0.420
** LDL-C/HDL-C ratio**	2.07 ± 0.82	2.06 ± 1.11	0.969
**Smoking history (%)**	10 (50.0)	90 (59.6)	0.359
**The reason for PCI**			0.084
** ACS (%)**	3 (15.0)	53 (35.1)	
** CCS (%)**	17 (85.0)	96 (63.6)	
**Number of CAC sites**	3.55 ± 2.24	2.38 ± 1.89	0.015
** >3 (%)**	12 (60)	63 (41.7)	0.122
** >4 (%)**	11 (55)	43 (28.5)	0.016
** >5 (%)**	9 (45)	25 (16.6)	0.003
**LMT calcification**	11 (55)	44 (29.1)	0.038

BMI: Body mass index; ICA: Internal carotid artery; PCI: Percutaneous coronary intervention; CAC: Coronary artery calcification; TG: Triglyceride; LDL-C: Low-density lipoprotein cholesterol; CI: Confidence interval; HDL-C: High-density lipoprotein cholesterol; SD: Standard deviation; ACS: Acute coronary syndrome; CCS: Chronic coronary syndrome; LMT: Left main trunk of coronary artery.

**Table 3:** Risk factors of future cerebrovascular events on multivariate analysis.

	Odds ratio	95% CI	*P* value
**Number of CAC sites**	1.417	1.092–1.839	0.009
**The reason for PCI (CCS vs. ACS)**	0.255	0.066–0.994	0.049

PCI: Percutaneous coronary intervention; CAC: Coronary artery calcification; CI: Confidence interval; ACS: Acute coronary syndrome; CCS: Chronic coronary syndrome.

The area under the curve was 0.684 (95% confidence interval, 0.513–0.855) for the number of CAC sites as a predictor of future CVEs (≥95th percentile). The cutoff value of the number of CAC sites for predicting future CVEs was 4.5 (sensitivity: 0.602, specificity: 0.743).

There was no significant cervical internal carotid artery stenosis for patients who occurred CVEs.

## Discussion

In this study, the incidence of CVEs after PCI in the chronic phase was approximately 10%. More CAC sites and post-PCI for CCS were independent predictors of future CVEs development. As with the traditional Agatston’s method, the new and easy method to assess CACs accurately reflected the risk of future CVEs. The incidence of post-PCI CVEs did not depend on the presence or absence of significant carotid artery stenosis.

Atherosclerosis is a significant cause of cardiovascular disease and stroke. It is characterized by the accumulation of lipids in arteries, the formation of plaques, and ultimately cardiovascular disease and stroke. Measuring atherosclerosis at the asymptomatic stage has important implications for understanding its progression in the clinical setting and may offer the potential for early interventions in a clinical and public health practice. Intervention before and during the preclinical stage of atherosclerosis may be an effective way to prevent cardiovascular disease and stroke. CAC is one of the risk factors for atherosclerosis, as well as hypertension, diabetes mellitus, dyslipidemia, smoking, overweight, and family history of heart disease [14]. In this study, some patients had systemic atherosclerosis. Systemic atherosclerosis may be the cause of CVEs after PCI in the chronic phase.

This study demonstrated the relationship between CAC and CVEs, which has previously been recognized as controversial. The Multi-Ethnic Study of Atherosclerosis showed that over 50% of carotid artery stenosis was associated with the presence of CAC [15]. The MESA study also showed that the CAC scores were an independent predictor of CVEs and improved incident CVEs to discriminate current stroke risk factors against the Framingham stroke risk scores [16]. In the Cardiovascular Health Study, the CCA-intima-media thickness was more strongly associated with stroke than CAC, but CAC was also a stroke predictor [17]. Kim et al. showed that moderate-to-extensive CAC was associated with the development of ischemic stroke (OR 1.72, 95% confidence interval 1.05–2.80, compared to subjects with no CAC) [18]. Lee et al. reported that the presence and extent of CAC were associated with ischemic stroke and were independent predictors [19]. The CAC score could predict stroke events, especially younger patients (under 65 years) and those in low-to-intermediate Framingham risk groups [20]. In contrast, in the Rotterdam study, there was no clear relationship between CAC and CVEs for a median follow-up of 3.5 years; calcification of the coronary, aortic arch and carotid arteries significantly predicted coronary heart disease, but not cerebrovascular events [21]. Stroke was significantly associated with carotid artery calcification, but not aortic arch or coronary artery calcification [22]. In this study, 11.6% of patients who received invasive coronary artery intervention suffered a cerebral infarction during a median follow-up period of 33 months. This study suggests that patients with more CAC sites may be most likely to benefit from earlier and more aggressive treatment of common risk factors to prevent CVEs and coronary artery disease.

Individuals with positive or increased CAC scores, calculated using traditional Agatston’s method, may be more likely to have an increased intracranial and extracranial vascular disease prevalence. Previous studies showed that individuals with higher CAC scores showed a significantly increased odds ratio (2.75–4.79) for intracranial arterial stenosis than individuals with lower CAC scores [23]. Other studies reported a relationship between CAC and cerebral small vessel diseases, including white matter lesions, asymptomatic lacunar infarction, and cerebral microbleeds. Small vessel diseases are thought to develop in association with the atherosclerotic state [24]. This study did not examine intracranial arterial stenosis. Some patients who experienced CVEs after PCI in the chronic phase may have some intracranial arterial stenosis.

In this study, post-PCI for CCS was also an independent predictor of future CVEs development.

In the early stage of arteriosclerosis, plaques are formed by intimal thickening, lipid deposition, and macrophage infiltration. In this stage, the vascular lumen is preserved by positive remodeling of the outer diameter. Further growth of plaques and compensatory insufficiency narrows the vascular lumen. Severe stenosis causes chronic coronary disease. Inflammation plays a vital role in the development and progression of this arteriosclerosis [25].

A lipid-rich necrotic core contains cholesterin crystals and inflammatory cell infiltration, and a thin fibrous fragile capsule covers this core. In this process, a vulnerable plaque is formed. If this vulnerable plaque ruptures, ACS occurs due to the thrombus formation in the coronary artery [26].

This difference between ACS and CCS may influence the future CVEs development.

Although we consider our findings to be valid, our study has some limitations. First, the follow-up period was not long, and therefore, some patients may experience a stroke in the future. This may have led us to underestimate the risk of future stroke in this population. Second, our study did not evaluate intracranial arterial stenosis; therefore, the exact etiology of stroke in our study was not clear.

## Conclusions

In our study, the incidence of CVEs after PCI in the chronic phase was about 10%. More CAC sites and post-PCI for CCS were independent predictors of future CVE. The new and easy method to evaluate CAC, proposed in this study, accurately reflects the future risk of CVEs after PCI in the chronic phase. There was no significant cervical internal carotid artery stenosis for patients who occurred CVEs.

## Supporting information

S1 Data (XLSX)
